# HPV transcript expression affects cervical cancer response to chemoradiation

**DOI:** 10.1172/jci.insight.138734

**Published:** 2021-08-23

**Authors:** Fiona J. Ruiz, Matthew Inkman, Ramachandran Rashmi, Naoshad Muhammad, Nishanth Gabriel, Christopher A. Miller, Michael D. McLellan, Michael Goldstein, Stephanie Markovina, Perry W. Grigsby, Jin Zhang, Julie K. Schwarz

**Affiliations:** 1 Department of Radiation Oncology,; 2 Division of Biological and Biomedical Sciences Molecular Cell Biology,; 3 Institute for Informatics,; 4 McDonnell Genome Institute,; 5 Alvin J. Siteman Cancer Center,; 6 Division of Nuclear Medicine, Mallinckrodt Institute, and; 7 Department of Cell Biology and Physiology, Washington University School of Medicine, St. Louis, Missouri, USA.

**Keywords:** Oncology, Cervical cancer, Radiation therapy

## Abstract

Persistent HPV infection is causative for the majority of cervical cancer cases; however, current guidelines do not require HPV testing for newly diagnosed cervical cancer. Using an institutional cohort of 88 patients with cervical cancer treated uniformly with standard-of-care chemoradiation treatment (CRT) with prospectively collected clinical outcome data, we observed that patients with cervical tumors containing HPV genotypes other than HPV 16 have worse survival outcomes after CRT compared with patients with HPV 16^+^ tumors, consistent with previously published studies. Using RNA sequencing analysis, we quantified viral transcription efficiency and found higher levels of E6 and the alternative transcript E6*I in cervical tumors with HPV genotypes other than HPV 16. These findings were validated using whole transcriptome data from The Cancer Genome Atlas (*n* = 304). For the first time to our knowledge, transcript expression level of HPV E6*I was identified as a predictive biomarker of CRT outcome in our complete institutional data set (*n* = 88) and within the HPV 16^+^ subset (*n* = 36). In vitro characterization of HPV E6*I and E6 overexpression revealed that both induce CRT resistance through distinct mechanisms dependent upon p53–p21. Our findings suggest that high expression of E6*I and E6 may represent novel biomarkers of CRT efficacy, and these patients may benefit from alternative treatment strategies.

## Introduction

Cervical cancer is the fourth leading cause of cancer incidence and mortality in women worldwide (1). In 2021, a total of 14,480 new cervical cancer diagnoses and 4290 cervical cancer deaths are expected in the United States alone (2). Persistent HPV infection is causative for more than 91% of cervical cancer cases (3).Encoded within the HPV genome are 2 oncogenes that target key cell regulatory components. HPV E6 targets p53, and HPV E7 targets pRb and p130, which are integral components of the pRb-related transcriptional repressor DREAM complex (4–7). Through E6 and E7, the virus induces cell immortalization resulting in a hyperproliferative state that facilitates viral replication. High-risk HPV genotypes use alternative splicing to switch between transcription of HPV E6 to E7 (8). Although a few of the high-risk HPV genotypes are able to encode multiple E6 splice variants, all of the high-risk HPV genotypes encode the E6*I (hereinafter referred to as E6*) splice variant (9). This primary alternatively spliced variant uses the first 5′ alternative splice site in the E6 open reading frame to generate a truncated E6 splice variant, E6*, and the full-length E7 transcript (8). E6* has been previously reported to encode a functional protein that acts as an antagonist to full-length HPV E6 (10, 11). HPV infection itself, however, is not sufficient to induce cervical cancer (12). The hyperproliferative state associated with E6 and E7 expression results in the development of cervical interepithelial neoplasms (CIN) 1–3, which can spontaneously regress. Over time, if HPV infection persists, the uncontrolled replication of the infected cell leads to the accumulation of mutations that can further deregulate and reprogram the cell, leading to tumorigenesis.

Previous studies have demonstrated that HPV status and HPV genotype are both prognostic indicators in cervical cancer patients (13–18). Cervical cancer patients with undetectable levels of HPV in their tumors have poor progression-free survival (PFS) and overall survival (OS) outcomes compared with patients with HPV^+^ tumors (13, 14). However, this HPV^+^ survival benefit is not binary, and patients with HPV genotypes other than HPV 16 in their tumors have worse PFS and OS after treatment than patients with HPV 16 detected (HPV^16+^) tumors (15–18). This observation is also seen when HPV genotypes are grouped by viral clade, where patients with α7 clade–positive tumors, which includes HPV 18, have worse PFS and OS compared with patients with α9-positive tumors, which includes the HPV 16 genotype (16, 18). Since HPV status has been shown to correlate with patient outcomes, it could be used as a diagnostic biomarker to identify patients who are likely to fail the current standard-of-care treatment strategy. However, the molecular basis of non–HPV 16 genotypes (HPV^Other^) conferring worse prognosis is not well understood. Additionally, even within previously reported studies, the HPV 16 patient subsets have PFS and OS below 75%, indicating that — within this clinically benefiting group — there may be some patients who are likely to fail their standard-of-care treatment and would benefit from a more personalized treatment plan (15, 17). Therefore, identifying a viral diagnostic marker independent of HPV genotype may be beneficial. Currently, it is unknown whether expression of HPV alternative transcripts affects tumor response to chemoradiation. The primary purpose of this study was to investigate whether variation in the viral transcriptome between HPV^16+^ and HPV^Other^ cervical cancer tumors correlated with poor prognosis after standard-of-care chemoradiation treatment (CRT). A secondary goal was to determine how HPV transcript expression, including the expression of alternatively spliced forms of HPV E6, affects cellular response to standard-of-care CRT.

## Results

### HPV genotypes detected in cohort.

**Figure 1:**
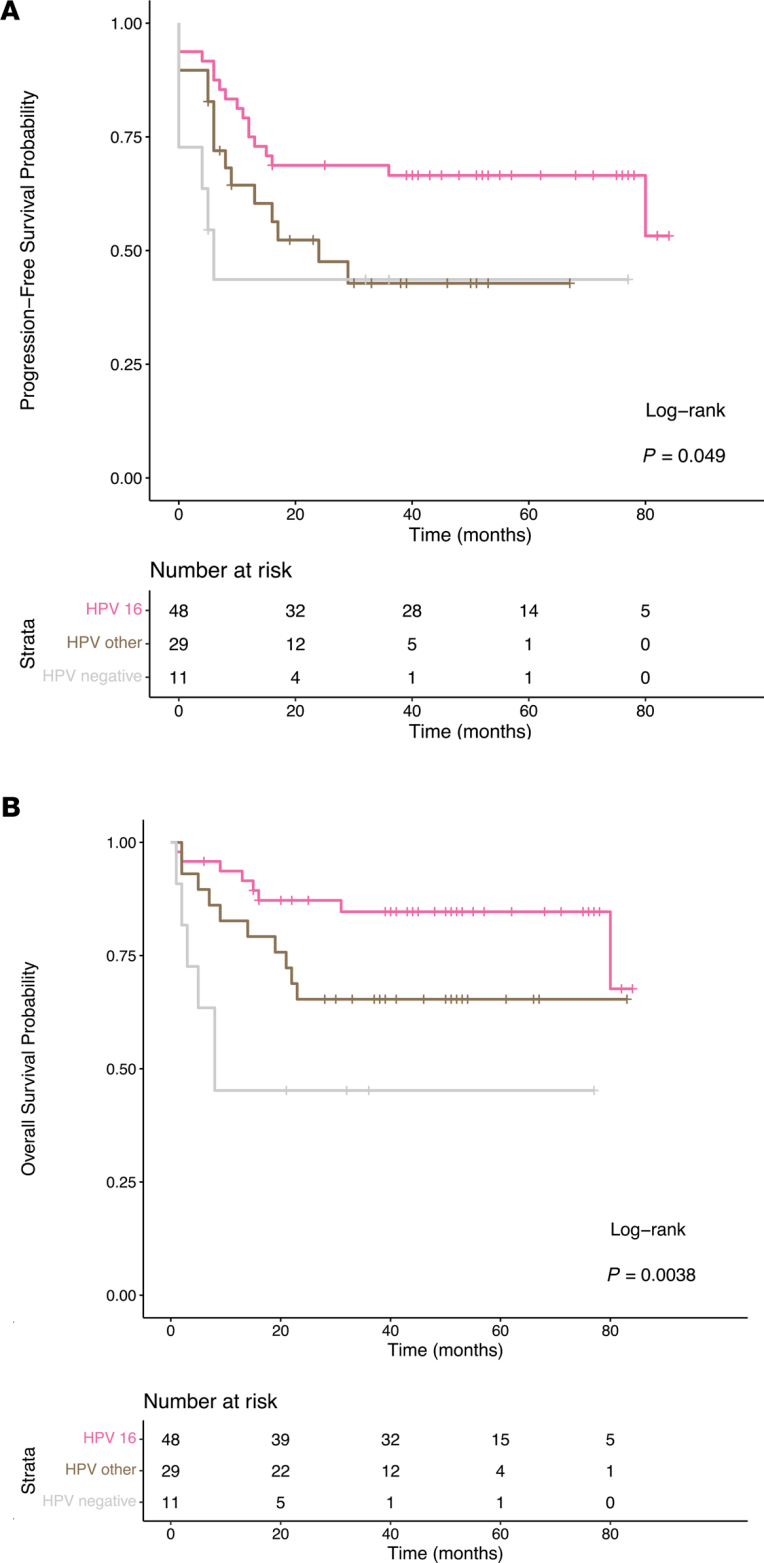
HPV genotype significantly stratifies patient outcomes. (**A** and **B**) Progression-free and overall survival curves stratified by HPV genotype (pink, HPV 16; brown, HPV other; gray, HPV undetected). Log-rank test was used to determine statistical significance, calculated using the survminer package in R version 3.5.2.

In our 88-patient cohort, 87.5% of patients had detectable HPV DNA, and patients with undetectable HPV DNA were excluded from the study. Patients with HPV^Other^ genotypes or multiple HPV genotypes detected were grouped together as HPV^Other^. Forty-eight patients had HPV^16+^ tumors, and 29 were HPV^Other^: 9 HPV-18, 4 HPV-33, 3 HPV-45, 3 HPV-52, 3 HPV-59, 1 HPV-31, 1 HPV-56, 1 HPV-58, 1-HPV 68a, 1 HPV-82, and 2 patients with multiple HPV genotypes detected (Table 1). Initial analysis focused on confirming whether prognostic differences based upon HPV genotype, clade, and viral integration observed in our institutional cohort was consistent with previously published studies. Patients with HPV^16+^ tumors had better PFS (*P* = 0.049) and OS (*P* = 0.004) compared with HPV^Other^ patients (Figure 1). Similarly, patients with α9-positive tumors (*n* = 57) had better PFS (*P* = 0.012) and OS (*P* = 0.084) compared with those with α7 clade (*n* =16) (Supplemental Figure 1, A and B; supplemental material available online with this article; https://doi.org/10.1172/jci.insight.138734DS1). Viral integration state was assessed using RNA sequencing (RNAseq) analysis, and integration events occurred in 70.3% of HPV^16+^ and 85.7% of HPV^Other^ tumors (Supplemental Figure 1E). Patients with integrated HPV had less favorable PFS (*P* = 0.067) and OS (*P* = 0.057) compared with patients with episomal HPV, although these results were not statistically significant (Supplemental Figure 1, C and D).

There were no significant differences in patient age at time of diagnosis, tumor staging, lymph node involvement, and metastasis at the time of diagnosis between HPV^16+^ and HPV^Other^ genotype patient groups (Table 2). Additionally, both patient groups had comparable radiation treatment intent and treatment completion (Table 2). Despite there being no difference in clinical characteristics or treatment strategies, patients with HPV^16+^ tumors had more favorable outcomes after chemoradiation compared with patients in the HPV^Other^ group. Patients with HPV^Other^ cervix tumors experienced more disease recurrence (*P* = 0.032) and increased mortality (*P* = 0.018) after CRT (Table 2).

### Comparison of viral transcription efficiency between HPV 16 and HPV other genotypes.

**Figure 2:**
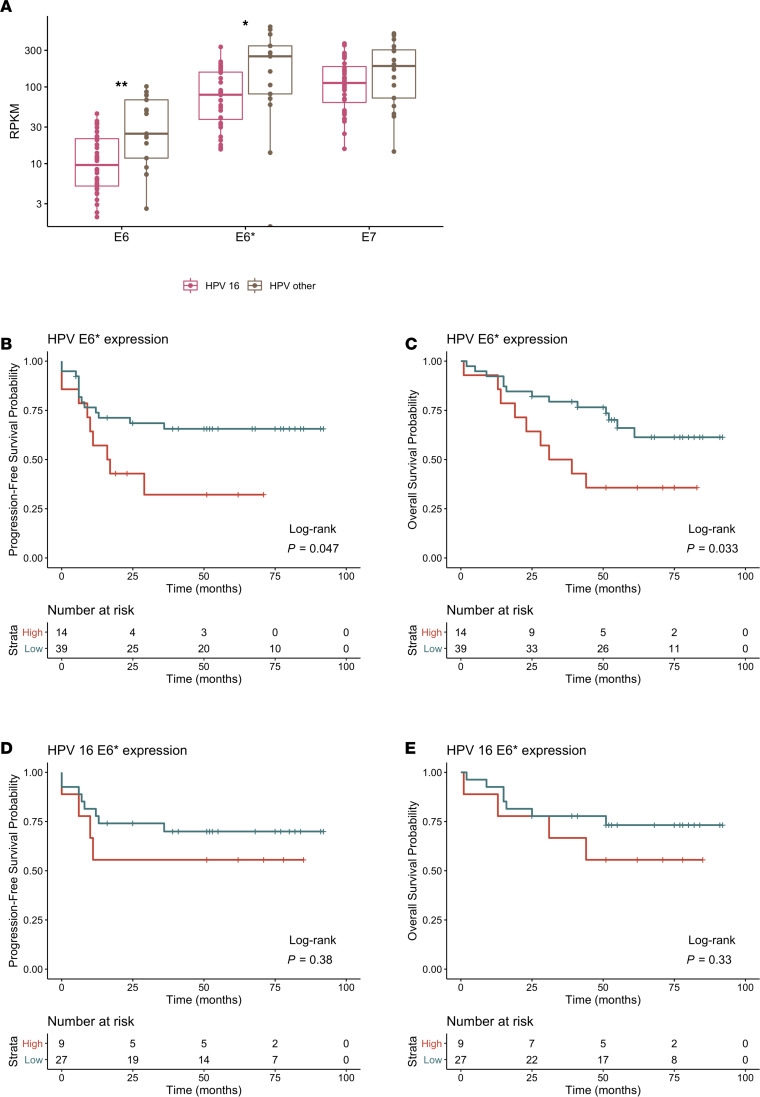
HPV E6, E6*I, and E7 transcript expression in Washington University in St. **Louis School of Medicine (WUSM) cohort.** (**A**) Comparison of HPV transcript expression between HPV 16 (pink) and non–HPV 16 ^+^ (brown) patient tumors (*P < 0.05, **P < 0.01; nonparametric Wilcoxon’s signed-rank test). (**B**–**E**) The full WUSM cohort (**B** and **C**) and the HPV 16 ^+^ patient subset (**D** and **E**) were stratified based upon their relative HPV E6*I transcript expression, where high > Q3 and low < Q3, and assessed for progression-free (**B** and **D**) and overall survival (**C** and **E**). Significance calculated by log rank test using the survminer package in R version 3.5.2.

RNA from 68 patient biopsies was isolated, and whole transcriptome sequencing, including all host and viral transcripts, was performed. Viral expression of E6, E6*, and E7 was quantified and compared between HPV^16+^ (*n* = 36) and HPV^Other^ (*n* = 17) patient groups. The HPV E6* and E7 transcripts were more highly expressed than HPV E6 in both HPV^16+^ and HPV^Other^ groups (Figure 2A). HPV^Other^ tumors had an average of 2.9-fold increase in HPV E6 (*P* = 0.0085) and 2.0-fold increase in E6* (*P* = 0.025) transcript expression compared with HPV^16+^ tumors. There was an average 1.5-fold increase in HPV E7 transcript expression of HPV^Other^ compared with HPV^16+^ tumors; however, this was not statistically significant (Figure 2A).

Similar observations were seen when HPV transcript expression was compared between integrated (*n* = 44) and episomal HPV (*n* = 14) patient groups (Supplemental Figure 1F).

We used publicly available TCGA cervical cancer data as a validation cohort for HPV transcript expression (https://portal.gdc.cancer.gov/). Similar to our cohort, 56% of tumor samples were HPV^16+^ and 43% were HPV^Other^ in the cervix TCGA cohort (Supplemental Figure 2A). There was no significant difference in the viral integration state between the HPV genotype groups (Supplemental Figure 2B). The HPV^Other^ patient group had an average fold increase of 3.0 for HPV E6 (*P* = 8.1 × 10^–12^), 2.2 for E6* (*P* = 2.4 × 10^–9^), and 1.4 for E7 (*P* = 0.0012) (Supplemental Figure 2C).

The relative expression levels of HPV viral transcripts were tested as predictive biomarkers for CRT outcome using prospectively collected clinical outcome data from our study’s uniformly treated patient cohort. HPV E6, E6*, and E7 transcript expression levels were grouped as high (reads per kilobase of transcript per million mapped reads [RPKM] > upper quartile) and low (RPKM < upper quartile). High HPV E6* expression was a poor prognostic indicator for PFS (*P* = 0.047) and OS (*P* = 0.033) after CRT (Figure 2, B and C). Interestingly, expression of HPV E6 or E7 alone was insufficient to significantly stratify patient survival outcomes (Supplemental Figure 3). To determine whether increased expression of HPV E6* could be used as an independent prognostic biomarker, we also analyzed the HPV^16+^ subset of patients alone (*n* = 36). HPV^16+^ patients with high HPV E6* expression again exhibited worse PFS (*P* = 0.38) and OS (*P* = 0.33) (Figure 2, D and E), although this did not reach the level of statistical significance.

### HPV transcript expression correlates in vitro with radiotherapy (RT) sensitivity.

To test our hypothesis in vitro, cervical cancer cell lines CaSki, SiHa, and SW756 were evaluated for their relative transcript expression of HPV E6, E6*, and E7 by quantitative PCR (qPCR). CaSki (HPV 16) had higher levels of E6, E6*, and E7 transcript expression compared with SiHa (HPV 16) and SW756 (HPV 18) cells (Supplemental Figure 4A). Next, we determined the sensitivity to radiation in these cervical cancer lines. The CaSki cell line with high viral transcript levels was more resistant to increasing doses of radiation compared with SiHa (4 Gy, *P* = 0.032) and SW756 (4 Gy, *P* = 0.017) cell lines with low transcript levels (Supplemental Figure 4B). Taken together, these results suggest that high viral transcript expression correlates with resistance to radiation in cervical cancer cells, consistent with our clinical observations.

**Figure 3:**
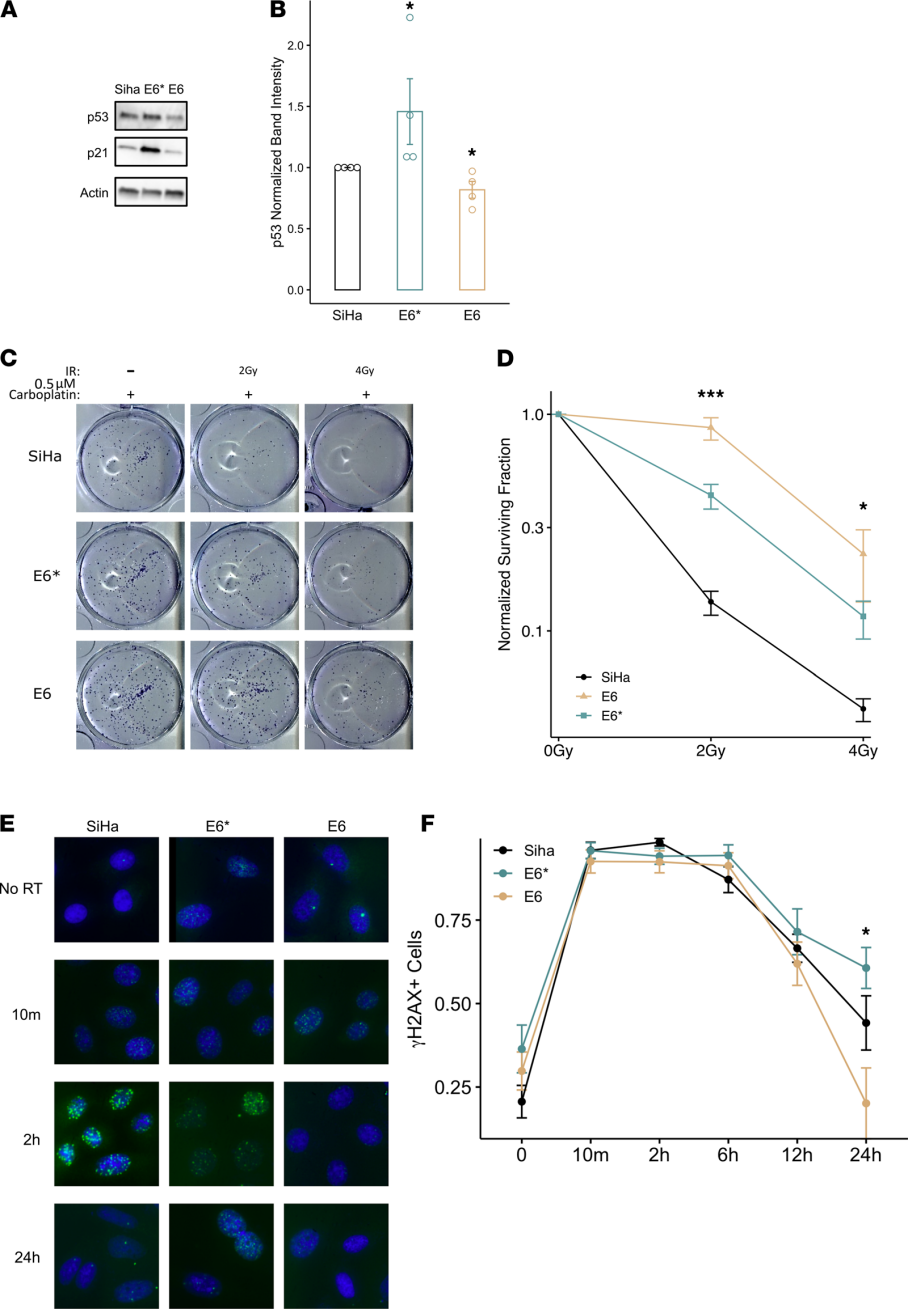
E6* and E6 overexpression effect on CRT response. (**A**) SiHa E6 and E6* expressing cell lines were quantified for p53 and p21 protein expression by Western blot. Representative blots; lanes run on the same gel but were not contiguous. Protein analysis was independently replicated 3 times. (**B**) Band quantification of p53 protein expression was performed on 3 independent blots (*P < 0.05, Wilcoxon’s signed-rank test). (**C** and **D**) Clonogenic survival was assessed by colony formation assay. (**C**) Representative images of colony formation assay shown. (**D**) Normalized surviving fraction of SiHa parental (black) versus SiHa E6* (blue) and SiHa E6 (brown) mean ± SEM. (**C** and **D**) Representative experiment shown with technical replicates *n* = 3 for each condition, one-way ANOVA test used. Experiment independently repeated 3 times). (**E** and **F**) DNA damage response was quantified using γH2AX p-S139 foci–positive cells. (**E**) Representative images of γH2AX foci (green) and nuclei stained by DAPI (blue) immunofluorescence of SiHa parental, SiHa E6*, and SiHa E6 cell lines. (**F**) Quantification of γH2AX foci–positive cells (>3 foci/nuclei) at 10 minutes, 2 hours, 6 hours, 12 hours, and 24 hours after 2Gy radiation treatment. Each condition had 4–10 random images quantified; data are representative of 2 independent experiments, using 1-way ANOVA.

Next, we wanted to remove any potentially confounding effects of HPV genotype and test whether alterations to HPV viral transcript expressions alone could induce resistance to CRT. The low HPV transcript expressing SiHa cell line was selected to evaluate the effect of increased E6 and E6* expression on cell sensitivity to CRT. HPV 16 E6 and E6* protein coding sequences were ligated into pCMV-6 mammalian expression vectors and transfected in SiHa cells, and primers specific to the vector HPV 16 E6 and E6* sequences were used to quantify vector specific transcript expression by qPCR (Supplemental Figure 5, A and B). E6 overexpression in SiHa cells resulted in a 26% reduction in baseline p53 levels, as expected (*P* = 0.0107). E6* overexpression in SiHa cells increased p53 protein expression compared with WT SiHa (fold change [FC] = 1.35, *P* = 0.0107) (Figure 3, A and B), consistent with previously published findings that E6* is an antagonist of E6-mediated p53 degradation (10).

### E6 and E6* expression affects cellular response to CRT.

We assessed the effect of E6 and E6* overexpression in SiHa cells on survival following CRT. Both, E6* and E6 overexpression decreased cell sensitivity to increasing doses of radiation treatment (Figure 3, C and D). Increased expression of E6* or E6 did not affect cell cycle progression following CRT (Supplemental Figure 6, A–F). We found that apoptosis was not the primary mechanism of cell death in the cervical cancer lines following CRT, although all cell lines were able to induce apoptotic cell death following etoposide treatment (Supplemental Figure 6, G–J). Kinetics of DNA damage induction and repair were determined by monitoring the formation and resolution of γH2AX foci after radiation. WT, as well as E6 and E6* overexpressing SiHa cells, showed similar levels of γH2AX foci 10 minutes after exposure to radiation. Twenty-four hours following irradiation, γH2AX were resolved in E6 expressing SiHa cells (*P* = 0.017), whereas WT cells and cells expressing E6* had residual γH2AX foci suggestive of unrepaired DNA breaks in the latter 2 lines (Figure 3, E and F).

### E6* overexpression induces p21-mediated cellular senescence.

**Figure 4:**
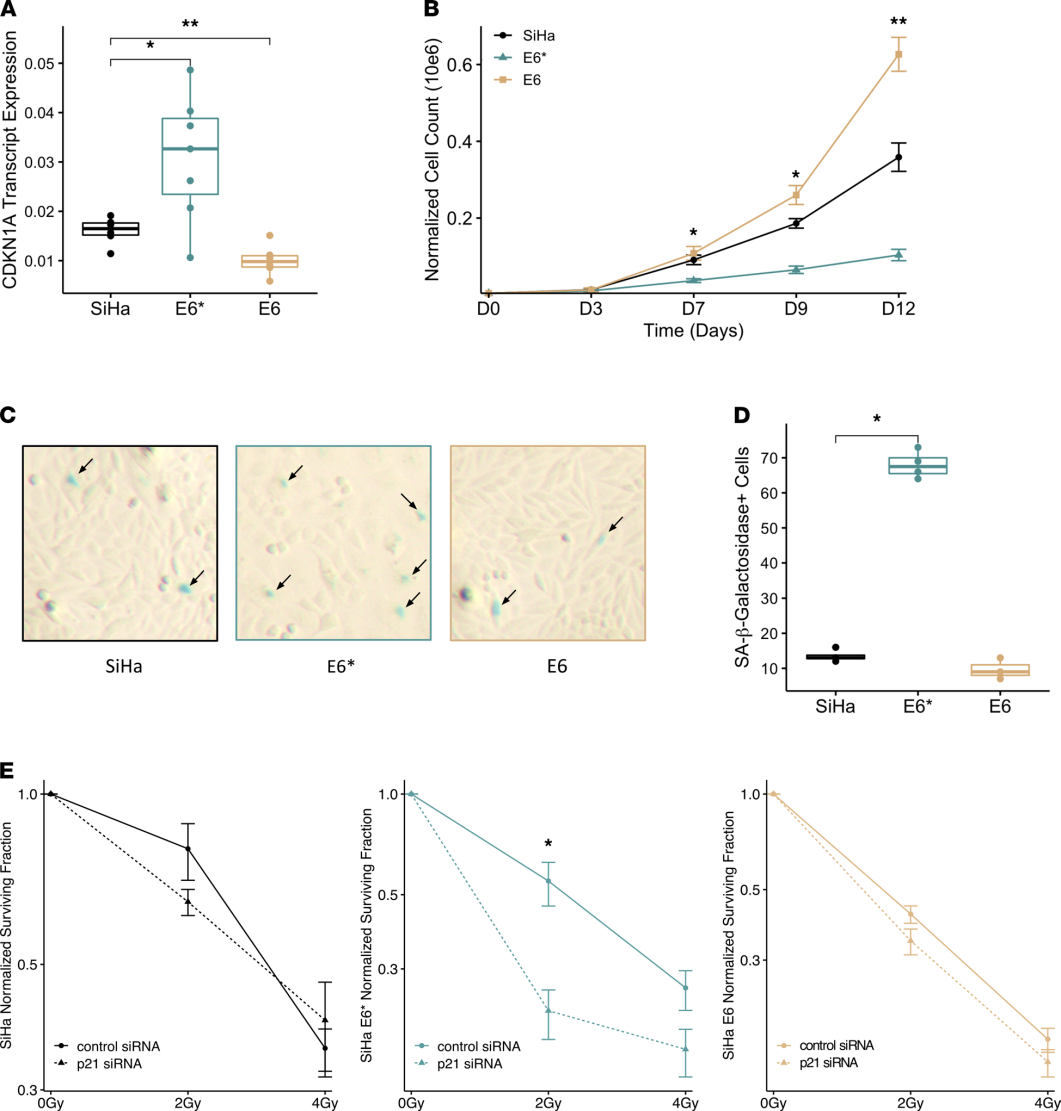
HPV E6* and E6 overexpression stabilizes p53 and p21 stabilization, leading to induction of cellular senescence. (**A**) CDKN1A (p21) expression was quantified using qPCR (normalized to GAPDH and ACTB transcript expression) (*n* = 6 biological replicates used for each, Student’s *t* test). (**B**) Proliferation assay of SiHa parental (black) versus SiHa E6* (blue) versus SiHa E6 (brown). Data representative from 3 independent experiments, mean ± SEM, 1-way ANOVA. (**C** and **D**) Representative images of SA-β-gal staining (arrow, SA-β-gal^+^) and quantification of SA-β-gal^+^ cells. Representative experiment shown from 25 random field images obtained per sample. Wilcoxon’s signed-rank test. *P < 0.05, **P < 0.01. Experiment was independently replicated 2 times.) (**E**) Normalized surviving fraction of each SiHa cell line treated with control siRNA versus a p21 targeting siRNA mean ± SEM. Representative experiment shown with technical replicates *n* = 3 for each condition. Student’s *t* test used. Experiment independently repeated 3 times.

To test whether the function of p53 as a transcription factor is affected by viral transcript expression, qPCR was performed for 6 p53 target genes. The SiHa E6* overexpressing cell line had higher *CDKN1A* (*P* = 0.021) and *NOXA* (*P* = 0.0089) transcript expression, and the SiHa E6 overexpressing cell line had lower *PUMA* (*P* = 0.045) transcript expression compared with the WT SiHa cell line (Figure 4A and Supplemental Figure 5, C and D). *BAX*, *GADD45A*, and *MDM2* transcript expression was not affected by either E6 or E6* overexpression (Supplemental Figure 5, E–G). *CDKN1A* encodes the p21 protein, which is a direct target of p53 regulation. SiHa E6* cells had 3.9-fold higher p21 protein expression compared with WT SiHa (*P* = 0.0028) (Figure 3A). The functionality of p21 was assessed in the 3 cell lines, including cellular proliferation and induction of cellular senescence. The p53 and p21 low SiHa E6 cell line had the fastest rate of proliferation, followed by WT SiHa. SiHa E6* overexpressing had the lowest proliferation rate (Figure 4B). High p53 and p21 expression can induce cellular senescence (19–21). To test whether increased p21 was associated with increased senescence in E6* expressing cervical cancer, SiHa (parent), SiHa E6*, and SiHa E6 overexpressing cells were plated and grown for 8 days and then stained for senescence-associated β-galactosidease (SA-β-gal) expression, and senescent cells were quantified. SiHa cells overexpressing E6* had more SA-β-gal^+^ cells compared with WT SiHa (*P* = 0.029) and SiHa E6 cell lines (*P* = 0.057) (Figure 4, C and D). There was no difference in SA-β-gal positivity between SiHa E6 and WT SiHa cells (*P* = 0.2). Increase in p21 is known to induce G1 cell cycle arrest (22); however, neither E6* or E6 overexpression induced a G1 arrest (Supplemental Figure 5, H and I). Additionally, previous reports have implied that an increase in p21 levels leads to transcriptional repression of cell cycle genes due to an interaction with the RB and DREAM complex (23). To test this, 5 DREAM target genes were selected, and their expression was evaluated across the cell lines; however, no significant alteration was found except for a decrease in *KIF23*, which was observed in both cell lines expressing p21 high, E6*, and p21 low, E6 (*P* = 0.01) (Supplemental Figure 5J). Both the parental SiHa cell line and the engineered SiHa E6* and SiHa E6 cell lines have HPV E7 expression intact, and E7 expression has been previously reported to abrogate both p21-dependent G1 cell cycle arrest and the p53–p21/DREAM pathway (24, 25).

### p21 knockdown sufficient to sensitize SiHa E6* to radiation treatment.

Lastly, we tested whether knockdown of p21 is sufficient to rescue treatment sensitivity in the p21 high E6* overexpressing cell line (Supplemental Figure 5, K and L). Transient knockdown of p21 in the parental SiHa and E6 overexpressing cell lines, which are low in p21 expression, did not have a significant impact on sensitivity to radiation treatment. In the p21 high E6* overexpressing cell line, transient knockdown of p21 led to an increase in cell sensitivity to increasing doses of radiation treatment (Figure 4E). Altogether, these findings indicate that overexpression of the p53 agonist E6* stabilizes p53 expression, leading to upregulation of p21 and subsequent induction of cellular senescence, and overall reduced sensitivity to CRT. This phenotype can be rescued by knockdown of p21 prior to radiation treatment.

## Discussion

Treatment for cervical cancer is currently guided by clinical staging, which includes the results of physical examination and whole-body imaging. In general, early-stage cervical cancers are managed by surgical resection, while locally advanced cases are treated with pelvic irradiation and the concurrent administration of cisplatin chemotherapy. While this treatment strategy is effective, 30%–50% of patients with locally advanced cervical cancer will fail CRT, necessitating the need to develop predictive biomarkers to assist in treatment planning. Current clinical guidelines do not require HPV testing of newly diagnosed cervical cancer. In this study, we demonstrate that expression of an HPV genotype other than HPV 16 is a predictive biomarker for a poor disease response to the standard-of-care CRT using a population of patients who were uniformly treated with standard-of-care CRT with prospectively collected clinical outcomes. Furthermore, using RNAseq, we demonstrated that cervical tumors with HPV^Other^ genotypes had higher viral transcript expression of HPV E6 and E6*. High HPV E6* expression was significantly associated with patient risk of developing disease recurrence and was a predictive biomarker of poor disease response following CRT. Interestingly, the prognostic value of high HPV E6* expression was also observed when restricting analysis to the HPV 16 patient subset, indicating that it may be a valuable genotype-independent predictive biomarker. These results suggest that more detailed analysis of HPV genotype and HPV-related gene expression could be used to identify cervical cancer patients at risk for treatment failure from standard-of-care chemoradiation.

These survival differences affected by the HPV types are not exclusive to cervical cancer. Approximately 20% of oropharyngeal squamous cell cancer (OPSCCs) are HPV related, and patients with HPV-related OPSCC have favorable outcomes after chemoradiation (26, 27). More recently, HPV genotype differences are being appreciated in HPV^+^ head and neck squamous cell carcinoma (HNSCC) where HPV 18 genotype has been identified as an independent poor prognostic factor of OS (28). In addition, expression of alternative transcripts such as HPV E1–E4 has been identified as a candidate biomarker for treatment resistance in HPV^+^ head and neck cancer (28). Based upon the results found in our study with prospectively collected tumor specimens and patient outcomes, as well as other previous studies, HPV genotype should be considered a predictive biomarker for outcome after chemoradiation. Furthermore, the association between HPV genotype and the expression of HPV-related genes, including full-length E6 and the alternatively spliced variant E6*, merit further study.

In this study, we focused on interrogating the relationship between HPV genotypes and viral transcript levels. Additionally, previous studies have shown that integrated HPV is a poor prognostic indicator in cervical cancer patients, although the biological mechanism to explain this clinical observation is poorly understood (29, 30). In our institutional cohort, 77% of HPV^16+^ patients had viral integration, whereas 83% of patients with HPV^Other^ tumors had viral integration (Supplemental Figure 1A). Similarly, in the cervix TCGA cohort, 70% of HPV^16+^ patient tumors had viral integration compared with 86% of HPV^Other^ tumors (Supplemental Figure 2A). For both independent patient cohorts, HPV^Other^ tumors tended to have a higher incidence of viral integration compared with patients with HPV^16+^ tumors. Additionally, in our institutional cohort, patients with HPV integrated tumors had worse PFS (*P* = 0.067) and OS (*P* = 0.057) after CRT; however, perhaps due to the small cohort size, these survival differences did not meet the threshold for significance (Supplemental Figure 1, B and C). Factors regulating HPV integration into the host genome are poorly understood. Host genome sites where HPV integration frequently occurs have been reported throughout the genome and are found within fragile sequence sites and areas of open chromatin (31). HPV integration into the host genome can result in transcription of viral-host fusion proteins and influence transcriptional activity of neighboring host genes via a pseudo-promoter effect (32). Using both our institutional cohort and validating with the cervix TCGA cohort, we found that the viral integration state also correlated with HPV transcript expression (Supplemental Figure 1D and Supplemental Figure 2D). As previously mentioned, for both our institutional and TCGA cervix cohorts, HPV^Other^ tumors had a higher incidence of viral integration, and the episomal group is primarily composed of patients with HPV^16+^ tumors (Supplemental Figure 1A and Supplemental Figure 2B). Taken together, this raises the question as to whether HPV^Other^ genotypes are more efficient in integrating their viral genome into the host genome, and perhaps this is why these patient tumors have higher viral transcript efficiency; however, additional in vitro work will need to be done to determine the relationship between HPV genotype, viral state and transcript expression.

In vitro characterization of high HPV E6* and E6 transcript expression revealed that increasing expression of either transcript was sufficient to induce CRT resistance. Each transcript induced treatment resistance through different mechanisms, but both involved differences in E6-related regulation of p53 and downstream p21 activation. Increasing full-length E6 expression inhibited p53 and p21 activity, resulting in enhanced efficiency of DNA repair of RT-induced lesions as evidenced by Figure 3, E and F. In contrast, high E6* expression inhibited E6-induced degradation of p53, resulting in p53 stabilization and downstream p21 activation, leading to induction of cellular senescence as shown in Figure 3A and Figure 4, C and D. Furthermore, we demonstrated that the decrease in treatment sensitivity observed in the high E6* expression cell line was dependent on the increase in p21 expression and could be sensitized to treatment following p21 knockdown (Figure 4E). Work is ongoing in our lab to use this model in vivo and investigate the pairing of novel drug therapies to sensitize high viral transcript expressing tumors to CRT.

Cellular senescence is a state in which cells no longer proliferate but are still viable. These cells alter the tumor microenvironment because of their senescence-associated secretory profile (SASP) and can reprogram cells of the microenvironment to a tumor-favorable phenotype (33). Senescent cells are nonproliferative and, therefore, have an intrinsic resistance to radiation treatment. However, an emerging group of chemotherapies are being developed to target and kill senescent cells (34). This new chemotherapy class should be tested for its efficacy in treating E6* overexpressing HPV transformed cell lines to determine whether targeting senescent cells will lead to better treatment efficacy following CRT (35, 36).

The enhanced DNA damage repair we observed in high E6 expressing cells should be further investigated to determine the mechanism of repair being affected by HPV E6 expression. In Figure 3, E and F, we show that HPV E6 overexpressing cells had enhanced phosphorylated-γH2AX foci clearance after radiation treatment compared with WT and E6* overexpressing cells. HPV E6 targets p53 for degradation, and — as we show in Figure 3A and Figure 4A — this decrease in p53 degradation coincides with a decrease in p21 transcript and protein expression. One of the functions of p21 is to inhibit PCNA, which is an essential component of DNA damage repair (37, 38). HPV E7 has been previously shown to upregulate PCNA expression (39). In our in vitro system, the SiHa E6 overexpressing cells express endogenous HPV 16 E7; therefore, the enhanced DNA repair that we find in this cell line following radiation treatment may be due to the synergy between inhibition of p21 by HPV E6 and the upregulation of PCNA by HPV E7. However, future studies will be required to identify the DNA damage repair pathway being affected by this alteration. Patients with high E6 expression could benefit from pairing radiation treatment with a DNA damage response inhibitor targeting the pathway affected by E6 overexpression.

In conclusion, using a population of patients uniformly treated with standard-of-care CRT with prospectively collected clinical outcome data, this study confirms HPV genotype and identifies, for the first time to our knowledge, HPV transcriptional efficiency as predictive biomarkers of poor patient outcomes after standard-of-care chemoradiation in cervical cancer. We validated the association between HPV genotype and viral transcript expression efficiency using the cervix TCGA cohort, which also demonstrated that HPV 16 ^+^ tumors indeed have lower transcriptional efficiency of HPV E6, E6*, and E7. Using cervical cancer cell lines engineered to overexpress E6 and E6* from HPV 16, we demonstrate that transcript variants of HPV E6 are associated with treatment resistance to standard-of-care CRT. While overexpression of full-length HPV E6 is associated with enhanced DNA repair, overexpression of HPV E6* is associated with tumor cell senescence. These results support more detailed examination of HPV genotype and viral transcript expression in patients with locally advanced cervical cancer and suggest that this information may be used in the future to identify patients at risk of CRT failure. In addition, these results provide important insights into treatment resistance mechanisms for HPV^+^ tumors.

## Methods

### Patients.

Patient tumor biopsies and blood were obtained prior to the initiation of therapy and stored at the Tissue Procurement Facility at Washington University in St. Louis School of Medicine. RT consisted of external beam irradiation and intracavitary brachytherapy, per institutional guidelines (Washington University in St. Louis School of Medicine), and concurrent cisplatin or carboplatin chemotherapy was also administered. Median follow-up time for patients alive at the time of last follow-up was 62 months (range, 25–92 months). Inclusion in downstream data analysis required detectable HPV in patient tumors. In the reporting of this data, we have adhered to REMARK guidelines (40).

### Clinical follow-up.

Patients were followed at the following time points after the completion of CRT: 6 weeks, 3 months, 6 months, 12 months, 18 months, 2 years, 3 years and 5 years. Physical examination including a pelvic exam was performed at each visit. Patients received posttreatment ^18^F-Flouro-deoxy-glucose positron emission tomography (FDG-PET) scans at 3 months after therapy to assess treatment response.

### HPV genotyping.

DNA isolated from 88 pretreatment tumors were sequenced using next-generation sequencing, and probes for the following HPV genotypes were used to identify the HPV genotypes present within the tumor: high-risk HPV-16, -18, -31, -33-, 35, -39, -45, -51, -52 -56, -58, and -59 genotypes and low-risk HPV-6b, -11, -26, -30, -53, -54, -57, -61, -66, -68a, -70, -72, -73, -82, -98, -99, -100, -104, -105, and -113. SAMtools idxstats (41) was used to extract the number of reads mapping to each HPV accession, and HPV positivity was defined as any sample with at least 100 HPV reads detected for one of the 32 genotypes. Patient groups were defined as patients with HPV^16+^, HPV^Other^, or no HPV detected in the sample (HPV undetected). Patients with undetectable HPV were excluded from study analysis.

### Viral transcriptome and integration state.

RNA was extracted, with 68 patients yielding at least the 0.5 μg of RNA with RIN > 7 sufficient for library preparation. RNAseq was performed for these patient samples using the Illumina HiSeq 3000. Raw sequencing reads were aligned to reference human and HPV viral genomes in GRCh38.d1.vd1 using STAR 2.7.0f, with chimSegmentMin set to 18 (42). STAR-generated chimeric junction files were parsed for reads that were chimeric between human and HPV references, and the corresponding alignment information and nucleotide sequences were extracted and manually inspected using BLAT and BLAST to verify viral integration. To obtain read counts for HPV E6, E6*, and E7, sequences were aligned against HPV reference genomes for both the E6 and E6* alternative splice using BWA 0.7.17-r1188. Numbers of junction spanning reads for WT E6 (*N_E6,J_*) and E6* (N_E6*,J_) were counted, as well as the number of reads aligned to E6 between the splice sites (*N_E6,b_*). The remaining *N_a_* reads align to the ranges over which E6 and E6* are identical. Total counts of E6 (*N_E6_*) and E6* (N_E6*_) reads were, therefore, obtained by assigning these reads to WT or E6* transcripts based on the proportions of each type of junction spanning read normalized by the size of the nt ranges from which such a read could arise, as in the following formulas:


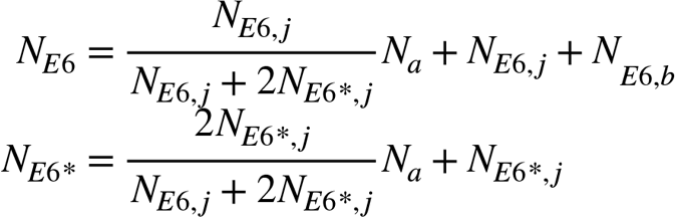


Counts of reads aligned to HPV E6, E6*, and E7 were then normalized by RPKM.

### TCGA viral transcriptome and integration state.

Viral gene expression and integration status was obtained for 304 TCGA-CESC primary tumor samples using a similar method to that employed for our institutional data. RNAseq reads were extracted and aligned to reference human and HPV viral genomes in GRCh38.d1.vd1 using STAR 2.7.0f with chimSegmentMin set to 18. Reads that were chimeric between human and HPV references were extracted, and samples with greater than 1 such chimera per million total reads were designated as positive for HPV viral integration. Normalized HPV E6, E6*, and E7 expression levels were also obtained as above.

### Engineering E6 and E6* overexpressing cell lines.

Cervical cancer cell lines were obtained from the American Type Culture collection (ATCC) and maintained in IMDM media (Invitrogen) with 10% heat-inactivated FBS and incubated at 37°C in 5% CO_2_. Mycoplasma testing was performed periodically to verify no infection. Last date of mycoplasma testing for cell lines used in this study was July 17, 2019, for SiHa and January 22, 2020, for CaSki and SW756. Experiments were performed on cell lines under passage 30. CaSki, SiHa, and SW756 were used to evaluate endogenous expression of HPV E6, E6*, and E7 transcripts and determine cell sensitivity to radiation treatment by alamarBlue (Thermo Fisher Scientific). SiHa had the lowest transcript expression for all HPV transcripts evaluated and was selected to engineer cell lines for the overexpression of HPV 16 E6* and E6. The mammalian expression vector pCMV-6 was used as the vector for exogenous HPV transcript expression. Origene VC101902 HPV 16 E6 open reading frame (ORF) was used as the codon optimized sequence for HPV 16 E6 minigene (Integrated DNA Technologies) and customized for HPV 16 E6* with an early stop codon inserted at cDNA position 151 (G > T) (Origene); sequences are provided in Supplementary File 1. HPV 16 E6 and E6* ORFs were restriction enzyme digested with 10 U/μL SgfI and 10 U/μL MluI, ligated into pCMV-6 vectors using T4 DNA ligase, and transformed into 5-α competent *E. coli* (New England Biosciences [NEB]) overnight and then selected using 25 μg/mL kanamycin LB agar plates. Midiprep of plasmids was performed using ZymoPure II Plasmid Midiprep Kit (ZymoPure) according to manufacturer’s protocol. Purified vectors were transfected into SiHa cells using Lipofectamine 3000 (Thermo Fisher Scientific). Transformed cells were G418 selected, and resulting cell pools were validated using qPCR for vector E6* and E6 sequence expression.

### Western blot.

Cells were lysed with Cell Lysis Buffer (Cell Signaling Technology), proteinase/phosphatase inhibitors and PMSF on ice for 30 minutes and then sonicated using the Bioruptor UCD-200 (Diagenode) on high for 5 minutes. NuPAGE LDS Sample Buffer (4×) (Invitrogen) and SDS buffer (2×) were added, samples were boiled at 95°C for 10 minutes and gel electrophoresed on 4-20% gradient gels (Mini-Protean TGX, Bio-Rad), transferred to PVDF blot, and blocked with 5% TBST milk. Blots were incubated with primary antibodies at 4°C overnight; anti-p21 (1:1000, Cell Signaling Technology [CST], 2947). HRP-conjugated secondary antibodies were incubated 1 hour at room temperature; anti-rabbit (1:4000, sc-2357), P53-HRP (1:1000, sc-126), and actin-HRP (1:5000 sc-47778) (Santa Cruz Biotechnology Inc.) were used with ECL chemiluminescent reagent (GE Healthcare Life Sciences). Blots were visualized and quantified using the Bio-Rad ChemiDoc MP imaging system and Image Lab software (Bio-Rad).

### qPCR.

Total RNA was isolated using the Trizol reagent according to manufacturer’s protocol (MilliporeSigma). Altogether, 1 μg of total RNA was used to synthesize cDNA using High Capacity RNA-to-cDNA (4387406, Thermo Fisher Scientific) kit per manufacturer’s protocol. Primers for HPV 16 and -18 native and vector sequences were synthesized by Integrated DNA Technologies, from reference sequences (8, 43–45) or as de novo sequences (Supplemental Table 1). Taqman probes were used for the p53 target genes: *CDKN1A*, *NOXA*, *PUMA*, *BAX*, *GADD45A*, and *MDM2* (Thermo Fisher Scientific). Probes for the DREAM target genes — *BIRC5*, *CDC25c*, *KIF23*, *MYBL2*, and *PLK4* — were obtained from Thermo Fisher Scientific. *GAPDH* and *ACTB* were used as reference genes. The qPCR was run at 50°C for 2 minutes, 95°C for 10 minutes, and 60°C for 1 minute using the 7900HT Fast Real-Time PCR System (Applied Biosystems). The specificity of the reaction was verified by melt curve analysis. Data processing was performed using SDS 2.4 software (Applied Biosystems), and the relative quantitation of each mRNA was performed using the comparative Ct method as described earlier (46).

### SA-β-gal assay.

Cells were plated and grown in culture for 7 days, harvested, and stained using the Senescence β-Galactosidase Staining Kit (9860, Cell Signaling Technology) per manufacturers protocol. SA-β-gal^+^ cells were quantified as number of cells per image frame with bright-field microscopy, in nonoverlapping regions.

### Proliferation, cell viability, and clonogenic survival.

To assess cellular proliferation, cells were plated at a density of 0.4 × 10^6^ cells per well and counted 3, 7, 9, and 12 days after plating on Vi-CELL cell counter (Beckman Coulter). For both cell viability and clonogenic survival, cells were treated with vehicle (0.01% DMSO) or 0.5 μM carboplatin 1 hour prior to radiation treatment (2 and 4 Gy). Cell viability was assessed 5 days after treatment using alamarBlue (DAL1025) (Thermo Fisher Scientific) per manufacturers protocol; viability was normalized to untreated samples. For colony formation assay, cells were treated as described, and 48 hours after treatment, cells were detached with 0.05% Trypsin and replated at 500 cells/well in a 6-well dish. Cells grew for 10–12 days, until untreated control wells reached colonies of > 50 cells. Plating efficiency (PE) was calculated as (number of colonies in control)/(500 cells seeded), and surviving fraction (SF) was calculated as (number of colonies in treated wells)/PE × (500 cells seeded). Cell lines were normalized to their own untreated control wells. For p21 knockdown colony formation assay, subconfluent (60%–70%) cells were transfected with 100 nM of p21 siRNA or control siRNA (Santa Cruz Biotechnology) using Lipofectamine RNAiMAX reagent (Invitrogen) in opti-MEM per manufacturer’s protocol. After 48 hours of siRNA transfection, transfected cells were irradiated and then processed as described.

### Immunofluorescence.

SiHa, E6, and E6* cells were seeded on chamber slides (Nunc Lab-Tek) and treated with single-fraction 2 Gy RT. Slides were fixed with 4% paraformaldehyde at 10 minutes, 2 hours, 6 hours, 12 hours, and 24 hours after irradiation. Cells were permeabilized and processed for immunofluorescence according to standard protocol. Slides were incubated with primary antibody 1:300 anti–phospho-Histone H2AX (Ser139, 20E3, Cell Signaling Technology) at 4°C overnight. Secondary antibody 1:500 Alexa Fluor 488 goat anti–rabbit IgG (Invitrogen, A11008) incubated 2 hours at room temperature. Slides were mounted using ProLong Gold Antifade Reagent with DAPI (Thermo Fisher Scientific). Slides were imaged with the Axio (Carl Zeiss) microscope.

### Flow cytometry.

Cell cycle and apoptosis were assayed using flow cytometry. For the following assays, cells were plated; the next day, they were treated with 0.5 μM carboplatin plus 4 Gy radiation. Cells were harvested at 24 and 48 hours following treatment. For cell cycle analysis, cells were washed and then stained with propidium iodide. For apoptosis, cells were washed then stained with annexin V and propidium iodide. Flow cytometry analysis was performed on MacQuant Analyzer and quantified on FlowJo. For each sample, 5000 events were collected.

### Statistics.

Tumor recurrence and OS were the primary endpoints of the study. Survival outcomes were measured from the completion of treatment. Recurrence was defined as new or progressive disease both locally within the pelvis and distantly outside the RT field. The Kaplan-Meier method and log rank test were used to determine differences in PFS and OS. R version 3.5.2 and the packages survminer and survival were used for the analysis. Wilcoxon’s signed-rank test was used to compare patient age between HPV cohorts. The χ^2^ and Fisher exact tests were used for all other patient categorical variables. The Wilcoxon’s signed-rank test and 2-tailed Student’s *t* tests were used for in vitro experiments. One-way ANOVA was used when comparing more than 2 groups. *P* less than 0.05 was set as the threshold for significance for all study outcomes.

### Study approval.

The study population included 88 patients prospectively enrolled into a tumor-banking protocol (January 2008 through July 2011). This study was approved by Washington University in St. Louis IRB, and all patients provided written informed consent for sequencing (approval no. 201105374). RNAseq data can be accessed at Gene Expression Omnibus (GEO) with accession no. GSE151666.

## Author contributions

SM, PG, and JKS contributed by writing the human studies protocol, enrolling patients, obtaining cervical tumor biopsies, prescribing and performing CRT, and prospectively collecting clinical outcome data. MDM and CAM performed HPV genotyping on the Washington University cohort tumor samples. FJR, MI, JZ, SM, NG, MG, RR, NM, and JKS conceptualized the study, designed experiments, and performed analyses. FJR, MI, and JZ performed data analysis of HPV genotypes, transcript expression, and integration status using Washington University and TCGA patient data. FJR, NM, and NG performed in vitro experiments. FJR and JKS prepared the manuscript. All authors contributed to final review of the manuscript.

## Supplementary Material

Supplemental data

Supplemental data set 1

## Figures and Tables

**Table 1:**
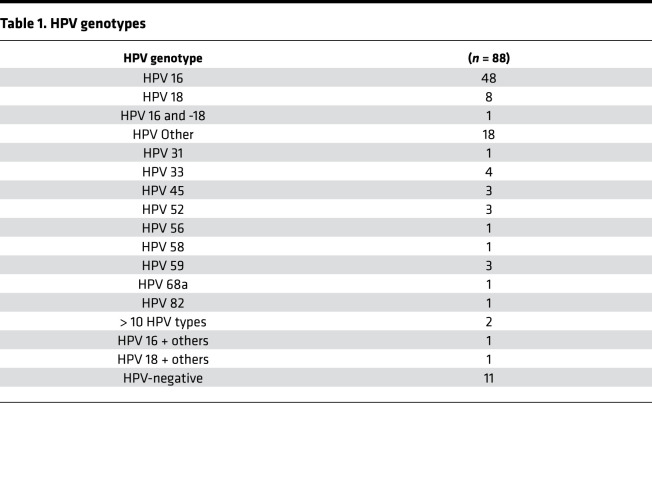
HPV genotypes

**Table 2:**
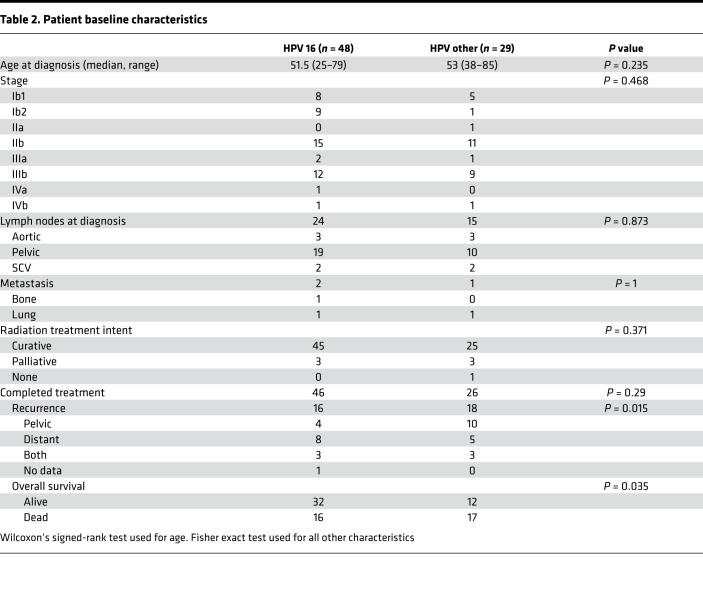
Patient baseline characteristics

## Version 1 07/13/2021

In-Press Preview

## Version 2 08/23/2021

Electronic publication
