# Supplementary material for: HPV transcript expression affects cervical cancer response to chemoradiation
Source: JCI Insight. 2021 Aug 23;6(16):e138734. doi: 10.1172/jci.insight.138734 (PMC8409981; doi:10.1172/jci.insight.138734)
Supplement: Supplemental data [file jciinsight-6-138734-s021.pdf]

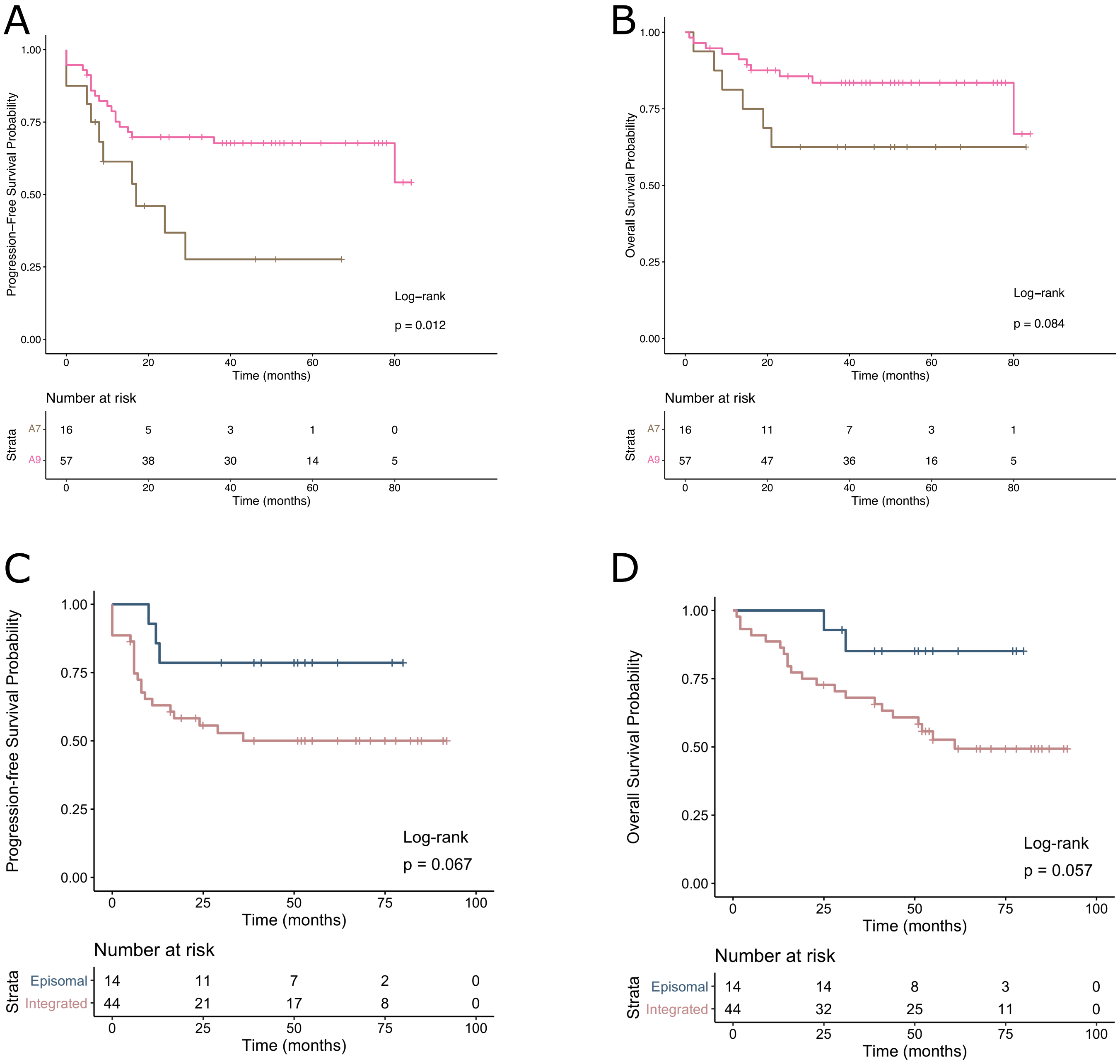

E

	HPV 16	HPV other	p-value
Episomal	11	3	0.2203
Integrated	26	18	
% Integrated	77%	83%	

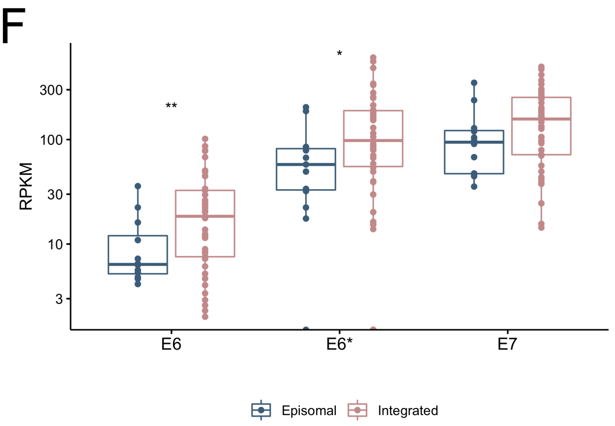

Supplementary Figure 1: Association of viral clade and state with patient outcomes and HPV transcript expression
Progression – free (A,C) and overall survival curves (B,D) stratified by viral clade (n = 36) and viral genome integration (n = 58). (A-D Log rank test was used to determine statistical significance, calculated using the survminer package in R version 3.5.2. E) Episomal and integrated samples by HPV genotype group (Fisher exact). F) HPV E6, E6* and E7 transcript expression (RPKM) by viral state (Wilcoxon signed-rank test).

A

TCGA HPV Genotype	n = 309
HPV 16	174
HPV 18	40
HPV other	93
NA	2

B

	HPV 16	HPV other	p-value
Episomal	40	23	0.2548
Integrated	134	110	
% Integrated	70%	86%	

C

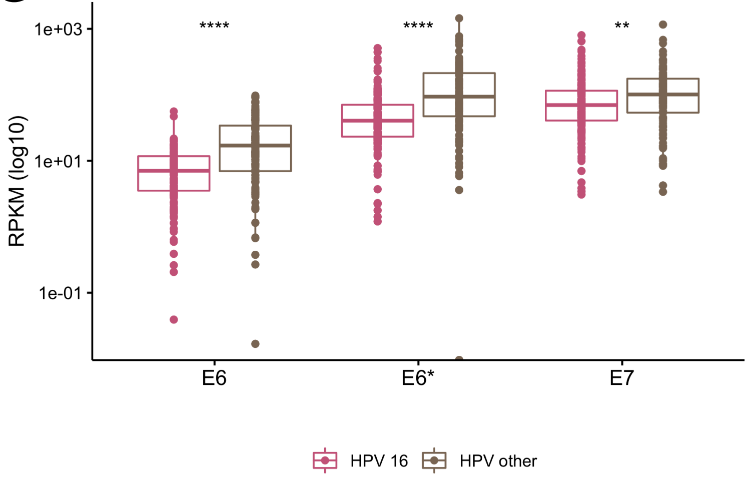

D

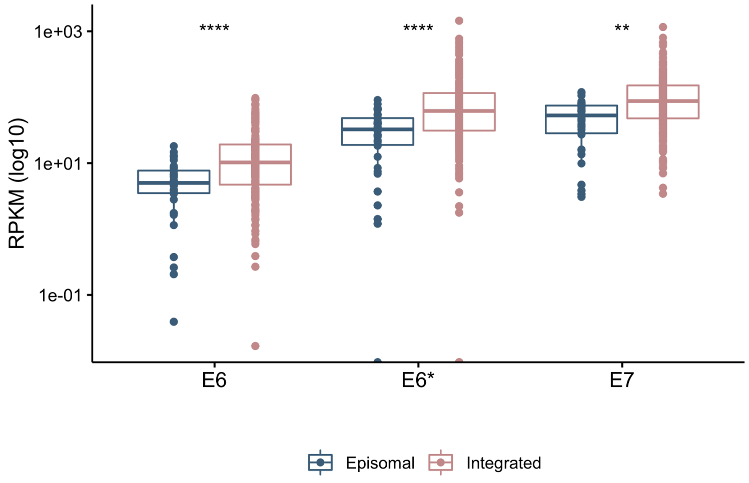

Supplementary Figure 2: TCGA viral state and HPV transcript expression

A) HPV genotypes and B) viral integration (chi-square test). C-D) HPV E6, E6* and E7 transcript expression (RPKM) by C)HPV genotype and D) viral state (Wilcoxon signed-rank test).

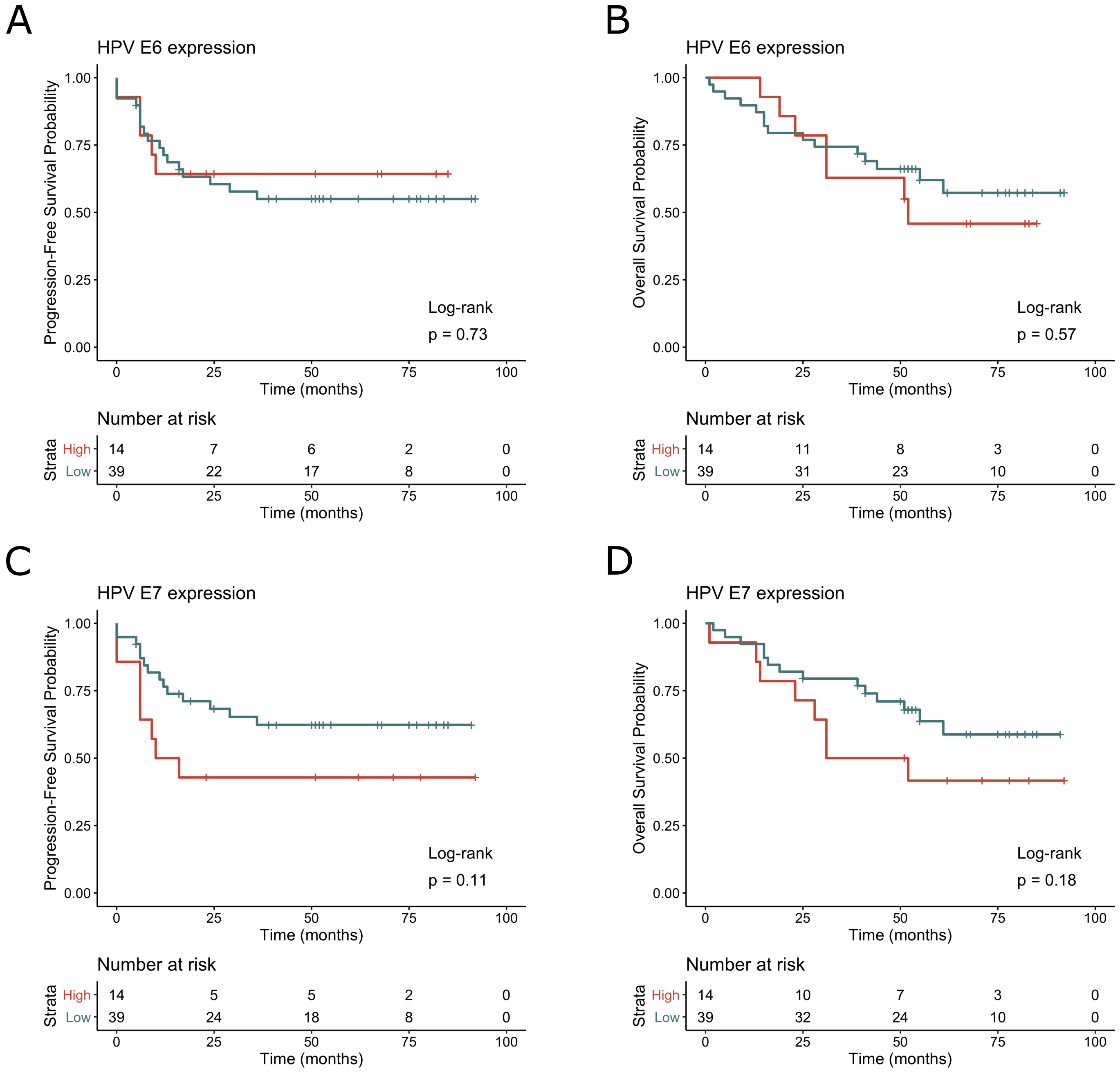

Supplementary Figure 3: Patient outcomes stratified by relative HPV transcript expression

Progression-free and overall survival was assessed for patients grouped based upon relative HPV transcript expression, high > Q3 and low < Q3 for A-B) HPV E6, and C-D) HPV E7 (Log rank test was used to determine statistical significance, calculated using the survminer package in R version 3.5.2)

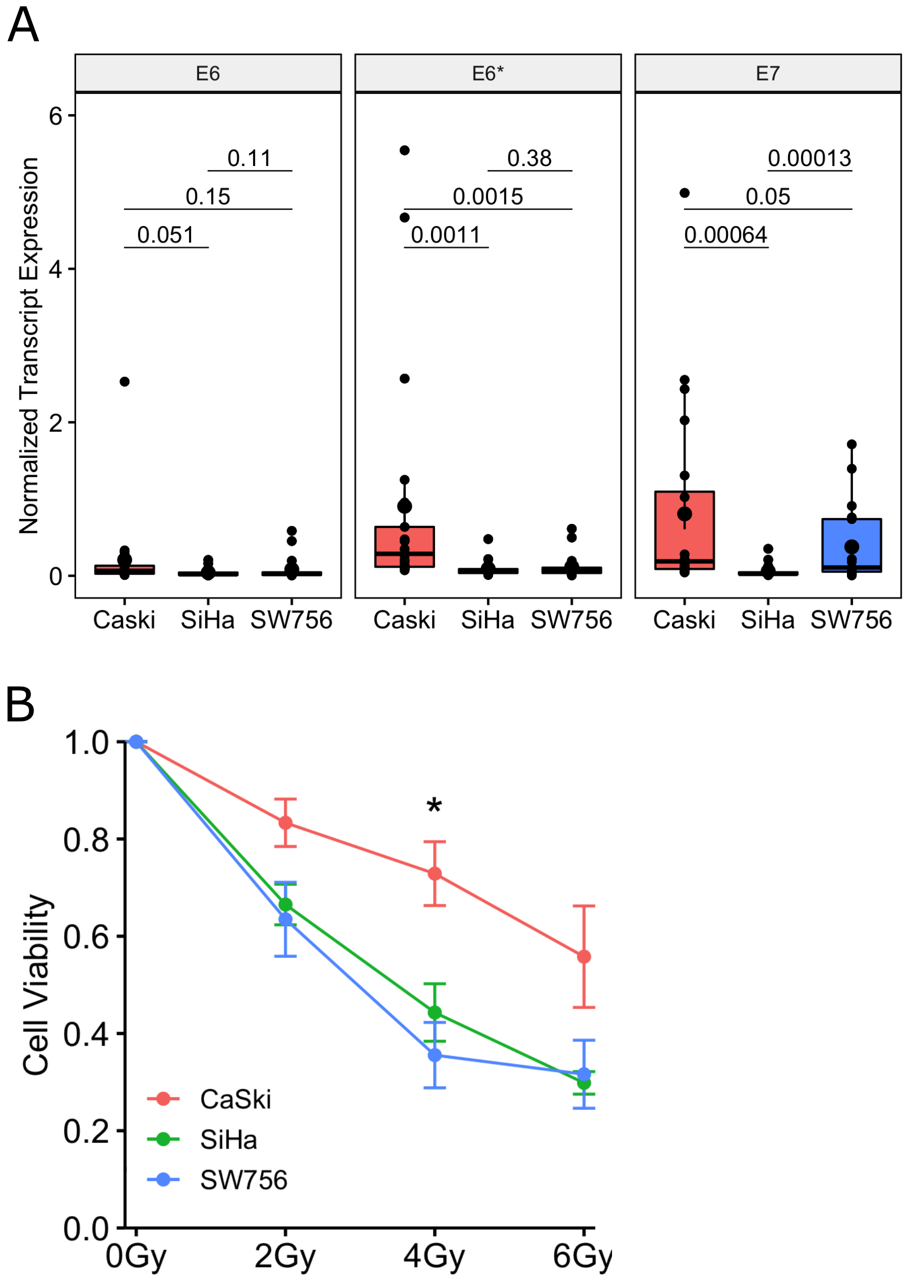

Supplementary Figure 4: Cervix cancer cell lines transcript expression and sensitivity to radiation treatment

A) CaSki, SiHa and SW756 relative HPV transcript expression by qRT-PCR (Normalized to GAPDH and ACTB transcript expression). (Data from 3 independent experiments, students t-test) B) Cell line sensitivity to increasing doses of radiation treatment assessed by alamarBlue. (Mean ± SEM, data from 3 independent experiments, one-way ANOVA).

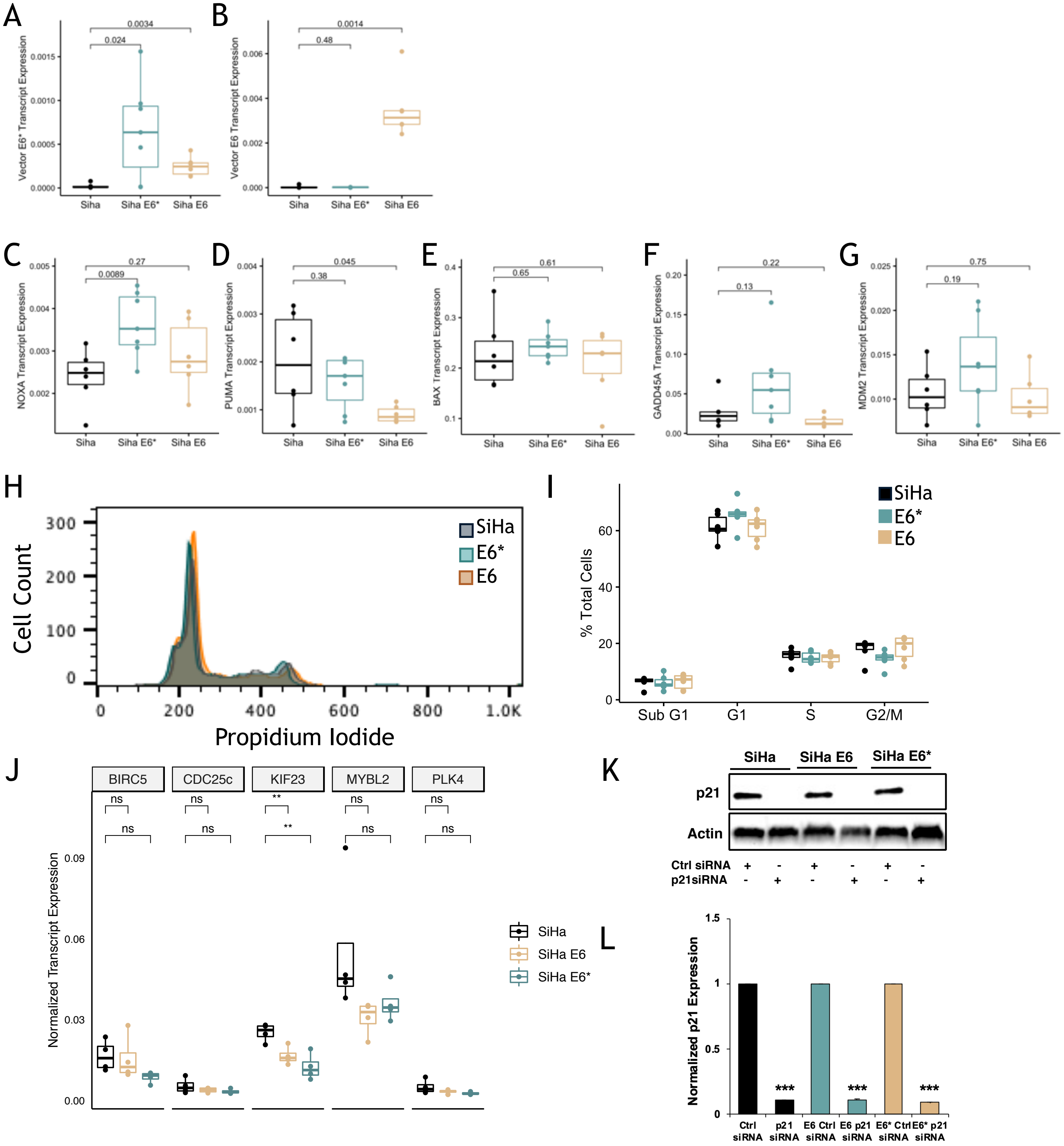

Supplementary Figure 5: Generation of HPV E6* and E6 overexpressing cell lines and their effect on p53 and p21

A,B) HPV vector specific expression of HPV 16 E6* and E6 across the cell lines. C-G) p53 target gene expression. (A-G Normalized to GAPDH and ACTB transcript expression, n = 6, students t-test). H-I) Basal cell cycle phase and quantification assessed by flow cytometry, no significant differences were observed across the cell lines tested (n = 3, one-way ANOVA). J) DREAM target gene expression (Normalized to GAPDH, n = 3, students t-test). K-L) Transient siRNA knockdown of p21 across the cell lines. (Normalized to Actin, lanes run on same gel but were not contiguous. n = 3, students t-test).

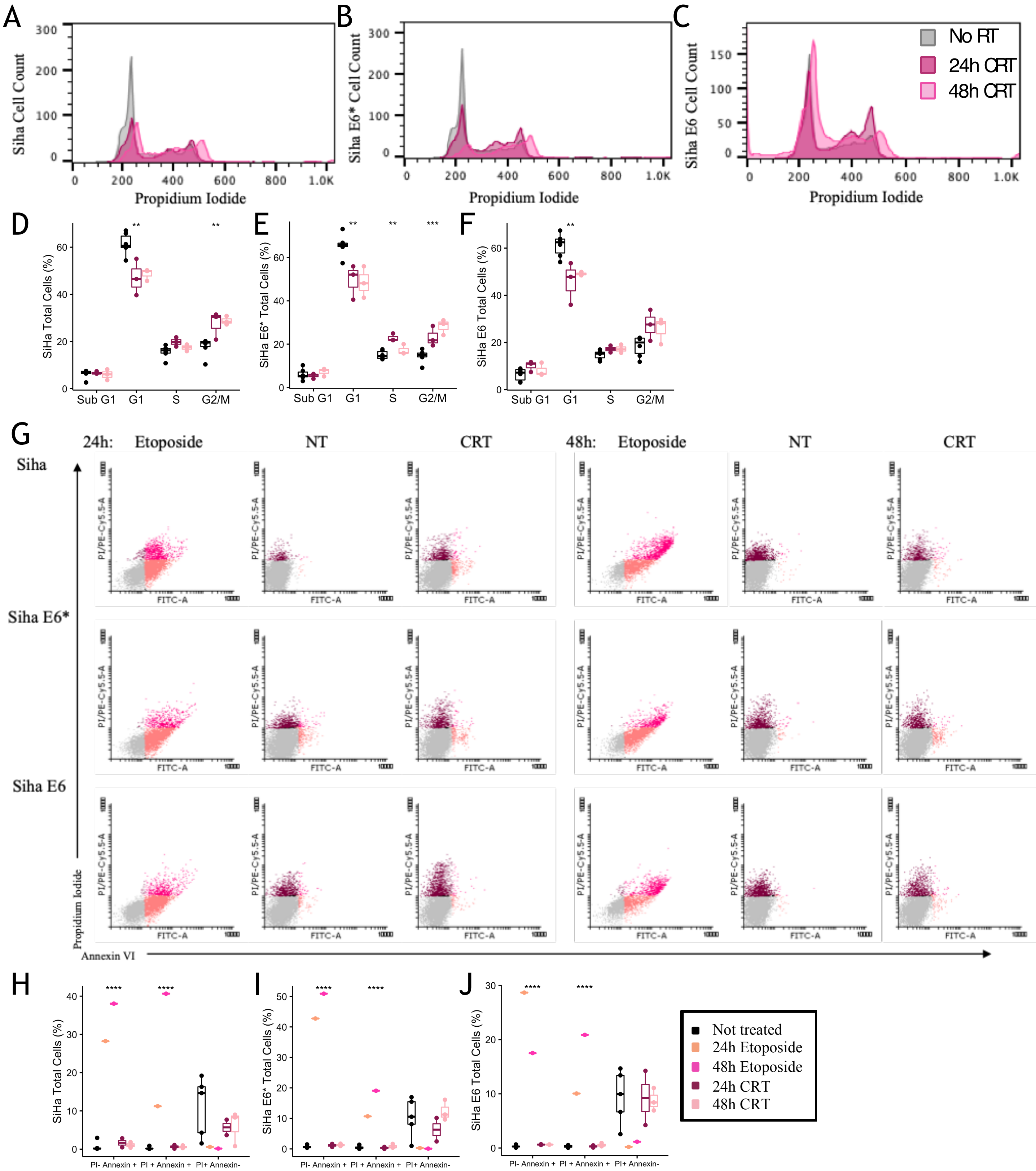

Supplementary Figure 6: E6* and E6 overexpression does not alter G2/M cell cycle arrest following CRT treatment and apoptosis not cell death mechanism post-CRT treatment

A-F) Cell cycle analysis by flow 24 and 48hrs after 0.5μM carboplatin and 4Gy radiation treatment. (dark pink = 24h, light pink = 48h). Representative flow and quantification of SiHa, SiHa E6* and SiHa E6 (n =3, one-way ANOVA). G-J) Apoptosis analysis by flow 24 and 48hrs after 0.5μM carboplatin and 4Gy radiation treatment. (dark pink = 24h, light pink = 48h). G) Representative flow plots of Annexin V and Propidium Iodide staining in SiHa, SiHa E6* and SiHa E6 using etoposide as a positive control for apoptosis, H-J) SiHa, SiHa E6* and SiHa E6 quantified staining (n = 3, one-way ANOVA).

Supplementary Table 1: HPV E6, E6* and E7 primer sequences

Native Sequences				
Genotype	Gene		Primer Sequence	Reference
HPV 16	E6	F	5’ - GCGACGTGAGGTATATGACTTT	
		R	5’ - TCAGGACACAGTGGCTTTT	7
	E6*	F	5’ - ACTGCAATGTTTCAGGACCCA	7
		R	5’ - TAATACACCTCACGTCGCAG	
	E7	F	5’ - AAGTGTGACTCTACGCTTCGGTT	34
		R	5’ - GCCCATTAACAGGTCTTCCAAA	
HPV 18	E6	F	5’ - ATCCAACACGGCGACCCTAC	35
		R	5’ - GCACCGCAGGCACCTTATTA	
	E6*	F	5’ - TTGGAACTTACAGAGGTGCC	
		R	5’ - CGAATGGCACTGGCCTCTATAG	36[a]
	E7	F	5’ - TAATCATCAACATTTACCAGCCCG	36
		R	5’ - CGTCTGCTGAGCTTTCTACTACTA	

Vector Sequences			
Genotype	Gene		Primer Sequence
HPV 16	E6	F	5’ - GAAGTGTACGATTTCGCTT
		R	5’ - GTGAGTAACAATAGTGCCT
	E6*	F	5’ - GTGTACTAATTCGCTTTCAG
		R	5’ - GTGAGTAACAATAGTGCCT

[a]Adapted from HPV 18 E6 R primer
