# Supplementary material for: HPV transcript expression affects cervical cancer response to chemoradiation
Source: JCI Insight. 2021 Aug 23;6(16):e138734. doi: 10.1172/jci.insight.138734 (PMC8409981; doi:10.1172/jci.insight.138734)
Supplement: Supplemental data set 1 [file jciinsight-6-138734-s022.pdf]

HPV 16 E6 and E6* vector sequences:

HPV 16 E6 ORF
GCGATCGCCATGCACCAGAAACGAACCGCTATGTTTCAGGATCCTCAGGAGAGACC
TCGCAAGCTCCCTCAGCTGTGCACTGAGCTCCAGACGACCATTCACGATATCATCCT
TGAGTGTGTGTATTGTAAACAGCAGCTGCTGAGACGGGAAGTGTACGATTTCGCTTT
CAGGGACCTCTGCATAGTCTACAGGGACGGAAACCCTTACGCCGTGTGCGACAAAT
GTCTGAAATTCTACTCCAAAATCTCTGAATATAGGCACTATTGTTACTCACTTTATGG
AACAACCCTGGAGCAGCAATATAATAAGCCTCTTTGCGACCTGCTGATTCGGTGCAT
CAATTGCCAGAAACCGCTCTGTCCAGAGGAGAAGCAGAGGCACCTGGACAAAAAAC
AGAGGTTTCATAATATACGGGGGCGGTGGACAGGCAGGTGTATGAGCTGCTGTCGG
AGTAGTCGAACAAGAAGGGAGACCCAGCTGACGCGTACGCGGCCGCTCGAGCAGA
AACTCATCTCAGAAGAGGATCTGGCAGCAAATGATATCCTGGATTACAAGGATGAC
GACGATAAG

HPV 16 E6* ORF (c.151 G>T)
GCGATCGCCATGCACCAGAAACGAACCGCTATGTTTCAGGATCCTCAGGAGAGACC
TCGCAAGCTCCCTCAGCTGTGCACTGAGCTCCAGACGACCATTCACGATATCATCCT
TGAGTGTGTGTATTGTAAACAGCAGCTGCTGAGACGGGAAGTGTAC**T**AATTCGCTTT
CAGGGACCTCTGCATAGTCTACAGGGACGGAAACCCTTACGCCGTGTGCGACAAAT
GTCTGAAATTCTACTCCAAAATCTCTGAATATAGGCACTATTGTTACTCACTTTATGG
AACAACCCTGGAGCAGCAATATAATAAGCCTCTTTGCGACCTGCTGATTCGGTGCAT
CAATTGCCAGAAACCGCTCTGTCCAGAGGAGAAGCAGAGGCACCTGGACAAAAAAC
AGAGGTTTCATAATATACGGGGGCGGTGGACAGGCAGGTGTATGAGCTGCTGTCGG
AGTAGTCGAACAAGAAGGGAGACCCAGCTGACGCGTACGCGGCCGCTCGAGCAGA
AACTCATCTCAGAAGAGGATCTGGCAGCAAATGATATCCTGGATTACAAGGATGAC
GACGATAAG
